# The Impact of Structural Variation on Alzheimer’s Disease in the Alzheimer’s Disease Sequencing Project

**DOI:** 10.21203/rs.3.rs-8562759/v1

**Published:** 2026-01-13

**Authors:** Songmi Lee, Adam C English, Gina M Peloso, Joshua C Bis, Eric Boerwinkle, Seung Hoan Choi, Nancy L Heard-Costa, Honghuang Lin, Rui Xia, Sudha Seshadri, Anita L Destefano, Myriam Fornage, Fritz J Sedlazeck

**Affiliations:** The University of Texas Health Science Center at Houston; Baylor College of Medicine; Boston University; University of Washington; The University of Texas Health Science Center at Houston; Boston University; Framingham Heart Study; University of Massachusetts Chan Medical School; The University of Texas Health Science Center at Houston; The University of Texas Health Science Center at San Antonio; Boston University; The University of Texas Health Science Center at Houston; Baylor College of Medicine

**Keywords:** Structural variation, Alzheimer’s Disease, genetics, association study, whole genome sequence

## Abstract

**Introduction:**

Structural variants (SV), genomic alterations spanning more than 50 base pairs, can significantly impact gene expression and protein function. However, their contribution to Alzheimer’s Disease (AD) remains poorly understood. Leveraging a novel SV calling pipeline, we identified SVs with high accuracy in a diverse sample of the Alzheimer’s Disease Sequencing Project (ADSP) and investigated the role of SVs in AD.

**Results:**

We analyzed SVs in 16,841 individuals from ADSP whole genome sequencing data using BioGraph, a semi-assembly-based method that employs graph-based representation for accurate SV detection. We identified 456,644 high-quality SVs, 65% of which were novel. Of these, 272,728 SVs directly impact genes, including 86 AD-related genes. Association analyses were performed within three ancestry groups, including 3,371 African (AFR), 6,327 European (EUR), and 2,126 Latin (LAT). Multiple deletions and insertions were observed in moderate to high linkage disequilibrium with known AD loci, including *TPCN1* and *TMEM106B*. In EUR, genome-wide association analysis identified two significant low-frequency deletions associated with AD, located in introns of *CCDC12* and *CCDC88B*, both encoding coiled-coil domain-containing proteins. Gene-based analyses further identified rare pathogenic SVs in several known AD genes, including *PSEN1* in LAT and *ABCA7* in AFR.

**Conclusions:**

Using a novel graph-based SV calling pipeline, we identified high-quality SVs across a large and ancestrally diverse cohort. Our analyses revealed both common and rare SVs associated with AD. These findings provide valuable insights into the genetic architecture of AD, emphasizing the value of including diverse populations in AD genomic studies.

## 1. Background

Alzheimer’s Disease (AD), characterized by progressive memory loss and declining cognitive function, is the most common form of dementia among older adults. It is estimated that almost 7 million Americans aged 65 and older are currently living with AD and this number is projected to double by 2060 [1, 2]. To date, there are few effective disease-modifying treatments or prevention strategies for AD, underscoring a critical need to better understand its etiology.

Genetic factors play a substantial role in AD, with a disease heritability estimated between 60–80% [3]. To date, genetic approaches have yielded important novel insights into AD etiology [4] and promise further advances in prevention, diagnosis, and treatment. Very rare highly-penetrant mutations have been identified in the Amyloid Beta Precursor Protein (*APP*), Presenilin 1 (*PSEN1*) and Presenilin 2 (*PSEN2*) genes that cause Mendelian forms of AD, typically with early onset [5]. More common alleles identified in large genome-wide association studies (GWAS) of sporadic late-onset AD have uncovered genes involved in cholesterol metabolism, endocytosis/phagocytosis, amyloid plaque and neurofibrillary tangle formation, and the innate immune system [6, 7]. Despite significant progress in understanding the genetic basis of AD, a substantial proportion of AD genetic architecture remains unknown [8]. Addressing this gap in knowledge requires a comprehensive characterization of all forms of genetic variation, beyond single nucleotide variants (SNVs), and including structural variation.

Structural variants (SVs) are typically defined as genomic alterations comprising 50 or more base pairs [9, 10]. These variants can be classified into five different types: insertions, deletions, inversions, duplications, and translocations. Compared to SNVs, SVs are numerically fewer but are larger in size, and therefore have a greater impact on DNA sequence and, consequently, on gene expression and function [11, 12]. SVs often occur in highly repetitive and polymorphic regions of the genome [13], making them challenging to detect with short-read DNA sequencing technology [10, 14]. Insertions are especially problematic and their role across diseases remains understudied [10]. Over the past decade, technological and methodological developments have improved SV detection from short-read whole genome sequence (WGS) data [10, 15], providing an opportunity to more comprehensively and accurately evaluate their impact on complex disease etiology.

In this study, we have implemented a novel SV calling method, BioGraph [16], on the WGS data from 16,841 subjects of the Alzheimer’s Disease Sequencing Project (ADSP, Release 3) and examined the association of the detected SVs with AD. Our study investigated the role of SVs in known AD loci, providing insights into the genetic architecture of AD. We also examined associations between common SVs across the genome and AD, and performed gene-based association testing to analyze rare SVs in AD.

## 2. Results

### 2.1. Identification of high-quality SVs using BioGraph in the ADSP

**Figure 1:**
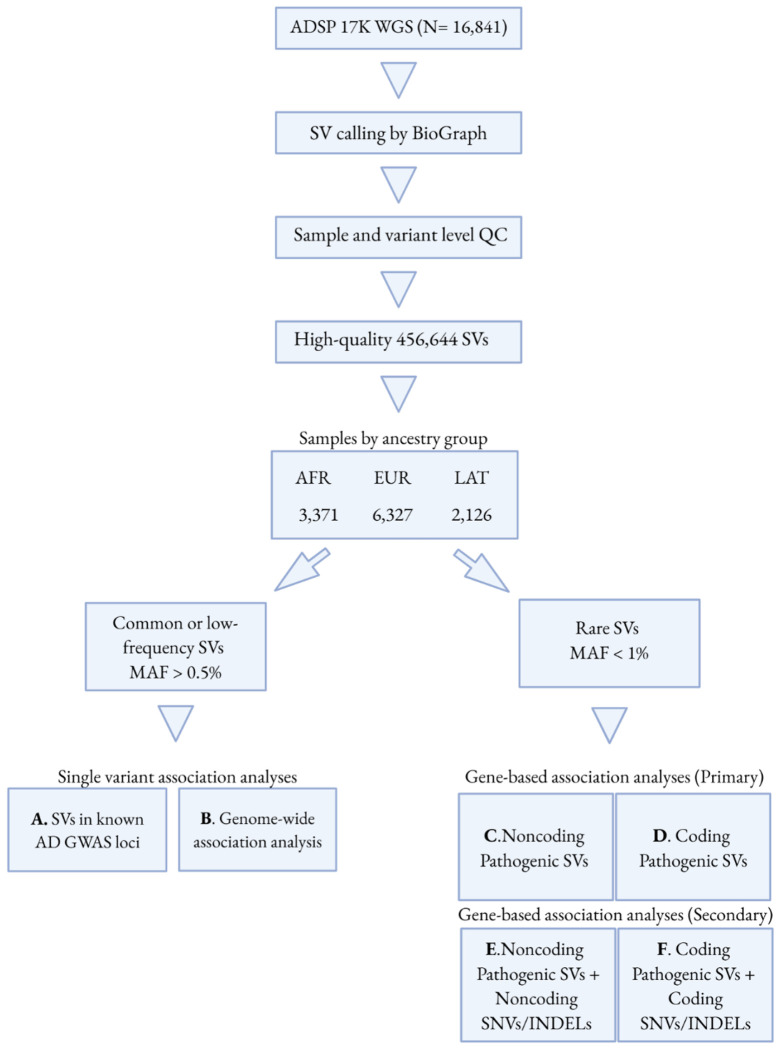
Study Overview. Whole-genome sequencing (WGS) data from 16,841 ADSP participants were analyzed using a novel SV calling pipeline (BioGraph), followed by extensive quality control at both sample and variant levels, resulting in 456,644 high-quality SVs. Samples were stratified into three ancestry groups: African (AFR), European (EUR), and LAT (Latin), based on genetic similarity. Common or low frequency SVs were evaluated using single variant association analyses, including analyses of SVs in known AD loci previously reported in genome-wide association study (GWAS) (A) as well as genome-wide association analyses (B). Rare SVs were assessed using gene-based association analyses as primary analyses, separately evaluating noncoding pathogenic SVs (C) and coding pathogenic SVs (D). Secondary gene-based analyses incorporated single-nucleotide variants (SNVs) and small insertion/deletions (INDELs) in combination with SVs to increase statistical power for noncoding (E) and coding (F) variants.

We implemented our novel method BioGraph [16] to generate a highly accurate SV call set from 16,841 WGS in the ADSP (Fig. 1). BioGraph is a unique approach to SV detection and genotyping that leverages reference guided assembly of short reads to improve the detection of SV. Additionally, BioGraph uses machine learning techniques to assign useful quality scores to the identified candidate SVs. Comparison of BioGraph performance in detecting SV with that of other SV calling tools, including Manta [17], Parliament2 [18], and Smoove [19], using benchmark data from the Genome In A Bottle HG002 Challenging Medically Relevant Regions [20] and Truvari [21] are described in detail in the **Supplementary Material**.

Our initial raw set of SV calls across the ADSP’s 16,841 sample set generated 1,019,035 SVs. We deployed novel sample-based filtering approaches to further ensure high quality and accuracy of SV calls across the samples. We first leveraged technical replicates present in the ADSP data. In total, there are 601 replicate samples derived from 283 unique individuals providing 428 replicate pairs. Using Truvari, we compared the SV calls between each replicate pair. First, we compared the quality score distributions of calls which were found consistently between replicates to those which were inconsistent (**Supplementary Fig. 1**). We determined a quality score threshold of 50 best segregates the calls by their consistency. These data and additional data examining the quality score’s relationship to False Positive and True Positive measurements in benchmarking experiments (**Supplementary Material**) suggest a minimum quality score of 50 should be applied for high quality calls. In total, 562,391 SVs were removed using this filter.

We also analyzed the similarity of the consistent calls between replicate pairs. Over 97% of consistent calls have a sequence/size similarity of 95% or greater, suggesting that SVs with ≥ 95% sequence and size similarity should be considered the same when performing inter-sample merging. Our rigorous QC procedure enabled us to assign pass and fail values across all SVs. Moreover, we collapsed and filtered 55.2% of the initially inferred SV and thus avoided many potential false positive or redundant alleles.

**Figure 2:**
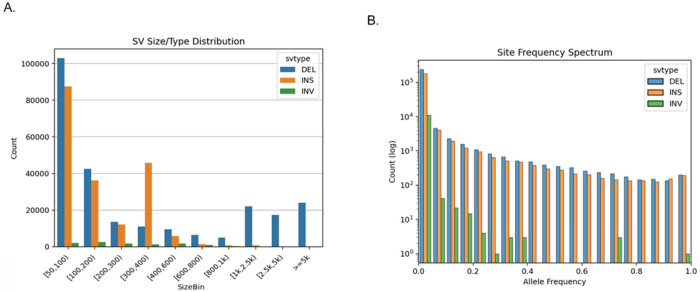
Size and allele frequency distributions of SVs in the ADSP (A) Distribution of SV sizes by variant type across the ADSP participants. SVs are grouped into size bins and colored by type: deletion (blue), insertion (orange), and inversion (green). The y-axis indicates the number of SVs in each size of category. (B) Site frequency spectrum of SVs by variant type, showing the distribution of allele frequencies for deletion (blue), insertion (orange), and inversion (green). The y-axis is shown on a logarithmic scale.

After QC, we identified 456,644 SVs, including 254,716 deletions (55.78%), 190,786 insertions (41.78%) and 11,142 inversions (2.44%). The distribution of SV size and type across the ADSP sample is shown in Fig. 2A. Most SVs identified were less than 5 kbp in length (94.74%) with a majority ranging from 50–100 bp (42.18%). We observed the expected ALU peak (~ 300 bp) for deletions but even a more prominent peak for insertions. The latter is due to our merging strategy from Truvari where the SV are only merged if their sequence similarity exceeds 95% in addition to type and length constraints [21].

The number of deletions and insertions were similar across most size categories with some exceptions. We identified fewer insertions in size categories larger than 1 kbp. This contrasts with other short read SV calling approaches where the number of insertions declines rapidly starting at 500 bp [10]. We also observed a size bias across inversions with only 210 inversions over 1 kbp detected. Figure 2B shows the site frequency spectrum (log scale). Overall, we observed that the insertions and deletions occur at similar frequencies and the majority of them (87.3%) have an allele frequency < 1%. Most of the inversions are singletons (70.7%) compared to deletions and insertions for which the proportion of singletons is 37.2% and 33.6%, respectively. These findings reflect the challenge to correctly infer inversions from short-read sequence data [14]. The singleton rate appears to vary with SV size, being lowest in smaller SVs (50–100 bp: 27.4% singletons) compared to midsize SVs (1 kbp-2.5 kbp: 51.1%) and large SVs (> 5 kbp: 60.7% singletons). We speculate that this is likely due to a combination of larger events accumulating mutations over time as well as larger events being less consistently discovered.

### 2.2. A total of 297,034 SVs are novel in the ADSP sample

We evaluated the overlap of our high-quality SV call set with previously reported SVs from major reference databases, including the Center for Common Disease Genomics (CCDG), Trans-omics for Precision Medicine (TOPMed), 1000Genomes Project (1KGP), and the Genome Aggregation Database (gnomAD). Only 34.9% of the SV identified in the ADSP overlapped with the reported SVs. The greatest overlap was found with gnomAD data (23.3% of ADSP SV calls), followed by 1KGP (19.4%). When investigating overlap by SV type, 18.9% of the overlapping SV were deletions, with the greatest overlap with gnomAD (12.33%); and 16% were insertions, with the greatest overlap with 1KGP (27.6%) rather than gnomaAD (26.3%). This may be due to tandem duplications in gnomAD reported as insertions in the 1KGP and our call sets, a common SV type swap [13].

We investigated the correlation of allele frequency among common overlapping SVs (MAF > 1%), and observed a strong overall concordance (r^2^ = 0.73, P < 0.01), underscoring the accuracy and reliability of the SV call set. As expected, the correlation was higher for deletions (r^2^ = 0.89, P < 0.01) compared to insertions (r^2^ = 0.63, P < 0.01). Interestingly, these correlations improved further when comparing the ADSP SV call set with external reference datasets. For example, the correlation of overlapping SVs in Biograph ADSP SV call sets and in gnomAD call sets was 0.94 for deletions and 0.82 for insertions.

Notably, many of the novel SVs identified in our study were within size ranges that were more effectively captured by BioGraph than methods relying solely on paired-end read distances. For example, the average deletion size in gnomAD was 7.4 kbp compared to 2.1 kbp in the Biograph ADSP data. For insertions, these numbers were 895 bp vs. 184 bp, respectively. These findings further highlight the strength of our dataset including SVs that may have been under-called by previous short-read studies.

### 2.3. Annotation and overlap of SVs with genes

We identified SVs that directly overlapped or were in close proximity (within 5 kbp) to gene sequences using SVAfotate [22]. All 456,644 SVs were annotated, of which 143,397 SVs (31.4%) mapped to intergenic regions, 131,988 SVs (28.9%) were reported in proximity of genes but not overlapping them directly, and 272,728 SVs (59.7%) directly impacted a gene. These 272,728 SVs mapped to a total of 30,333 (62.8%) genes, suggesting that they are impacting the same gene more than once across different individuals. The majority of these gene-impacting SVs were deletions (53.3%) followed by insertions (36.6%) and inversions (10.1%). The lower number of gene-impacting insertions compared to deletions is likely because insertions are measured as affecting only the direct base pair at which they are reported, while deletions span multiple base pairs on the reference. The majority (96.9%) of gene-impacting SVs were located within introns whereas 3.0% mapped to the 3’ untranslated region (UTR) and only 1.6% mapped to the 5’ UTR. The SVs mapped to UTR have a higher chance to impact regulatory function itself. Only 2.3% of SVs were directly overlapped coding sequences.

Among 86 AD genes reported by the ADSP Gene Verification Committee, 82 genes intersected 1,223 SVs. Most genes (71) had SVs within ± 5kbp as well as overlapping the gene body. Filtering to only common (AF ≥ 1%) SVs hitting non-intronic gene bodies left 69 variants over 30 genes (**Supplementary Table 1**). SVs of note included a 6,137bp deletion on *PRDM7* (AF = 2.4%), 4 tandem repeat expansions between 51bp and 98bp of a 12bp VNTR in *RBCK1*, and a 322bp deletion on *TMEM106B* with a frequency of 49.7%.

### 2.4. PCA and ancestry inference

We derived principal components from our SV data (N = 12,908) to account for possible population structure in the data. PC2 was associated with read length and demonstrated complete separation of samples as shown in **Supplementary Fig. 2**. To minimize confounding by batch effects in the association analyses, study participants were further restricted to those with a read-length of 150 (N = 11,890; 5,585 cases and 6,305 controls), which represents more than 90% of the sample. PCA analyses revealed similarities in results between PCs derived from SVs and those derived from SNVs (**Supplementary Fig. 3**). Based on the results of GrafPop (**Supplementary Fig. 4**), our study included 3,371 individuals of African (AFR) ancestry, 6,327 of European (EUR) ancestry, 2,126 of Latin (LAT) ancestry, and 66 participants that did not cluster with those three ancestry groups and were therefore excluded from subsequent analyses (**Supplementary Table 2**).

### 2.5. SVs in linkage disequilibrium (LD) with Alzheimer’s Disease known loci

Ancestry-specific LD analyses identified 9 SVs in EUR, 5 SVs in AFR, and 9 SVs in LAT that were in moderate or high LD (r^2^ = 0.43–0.99) with at least one of the SNPs previously identified in AD GWAS (**Supplementary Tables 3–5**). These SNPs did not exhibit strong associations with AD in our dataset, due to limited statistical power compared to the GWAS sample size in which they were discovered. Among the identified SVs, 4 SVs in EUR, 1 SV in AFR, and 1 SV in LAT showed suggestive evidence of association with AD (SV P-value < 0.1).

The strongest SV association with AD was observed in AFR, involving a 122-bp deletion in moderate LD (r^2^ = 0.46) with rs2633682 tagging the *ALCAM* locus (**Supplementary Table 4**). This SNP, previously associated with AD specifically in an African American population [23], showed a suggestive association with AD in our dataset (SNP P-value = 0.009). However, conditional analyses indicated that neither the SVs nor the SNP remained significant after adjusting for each other, suggesting non-independence of the signals at the *ALCAM* locus. In EUR, a similar trend was observed at the *ALCAM* locus, although the associations were weaker.

A 319-bp deletion in an intron of *TPCN1* was observed in all three ancestry groups and was in high LD (r^2^ = 0.97 in all groups) with the tagging SNP. In EUR and LAT, two deletions, including the 319-bp deletion, and one insertion were in moderate or high LD with a SNP tagging at the *TPCN1* locus (**Supplementary Tables 3 and 5**). Haplotype estimation analysis suggested that all detected SVs lie on the same haplotype as the AD risk allele at this locus (**Supplementary Fig. 5**). A 68-bp deletion located in an intron of *SLC8B1* were detected in EUR and LAT, with suggestive association with AD observed in EUR (SV P-value = 0.03). While the intronic SNP (rs6489896) tagging *TPCN1* has been previously associated with AD at genome-wide significance, *SLC8B1* has not. Additionally, a 322-bp Alu deletion in exon 8 and 3’ untranslated region of *TMEM106B* exhibited strong LDs with two tagging SNPs in EUR and LAT, and moderate LD in AFR.

Conditional analyses adjusting for the corresponding SNPs revealed that none of the SV associations remained (adjusted SV P-value > 0.1), indicating that the observed suggestive SV associations were not independent of SNPs in LD at those loci. However, in LAT (**Supplementary Table 5**), a SNP tagging *WNT3*/*MATP* locus remained significant (adjusted SNP P-value = 0.006) after conditioning on a 314-bp deletion in moderate LD (r^2^ = 0.68), suggesting that the SNP is independently associated with AD at this locus.

### 2.6. Genome-wide association of common or low-frequency SVs with Alzheimer’s Disease

For each ancestry group, we performed single variant association analyses of high-quality SVs with MAF > 0.5%, and in HWE (as defined in section 4.8). In total, we analyzed 28,942 SVs in AFR, 14,656 SVs in EUR, and 30,394 SVs in LAT (**Supplementary Table 6**). No SVs were significantly associated with AD in the AFR or LAT analyses, or in the meta-analyses. In EUR, two deletions were significantly associated with AD at the Bonferroni-corrected threshold and were observed exclusively in this ancestry group (Table 1; **Supplementary Fig. 6**). Both deletions mapped to introns of genes encoding coiled-coil domain containing proteins.

### 2.7. Gene-based association of rare SVs and SNVs with Alzheimer’s Disease

We performed gene-based analyses to test association between aggregated rare SVs and AD. In the primary gene-based association analyses, two analyses were carried out based on the SV types: coding SVs (**Supplementary Fig. 7**) and noncoding SVs (**Supplementary Fig. 8**). No genes reached genome-wide significant or suggestive significance thresholds in either analysis.

In the secondary analyses, we conducted gene-based analyses to assess association between AD and aggregates of rare SVs and rare SNVs/INDELs. Coding variant analyses were performed using five categories of SNVs/INDELs, combined with pathogenic coding SVs (**Supplementary Fig. 9**), while non-coding variant analyses included eight categories of SNVs/INDELs in combination with pathogenic non-coding SVs (**Supplementary Fig. 10**).

In the secondary coding variant analyses, we identified *PSEN1* as being suggestively associated with AD in LAT when aggregating SVs and SNVs/INDELs classified as pLoF and disruptive missense (P = 1.9E-07), disruptive missense (P = 1.9E-07), or missense (P = 8.6E-07) variants (Table 2A). For this region, we observed two deletions and two insertions, all more frequent or exclusively observed in AD controls (**Supplementary Table 7**). Conditional analyses on the aggregates of SNVs/INDELs in *PSEN1* indicated that the four pathogenic coding SVs were independently associated with AD (adjusted P-values < 0.05). No SVs were detected for the *SMOC1* and *ACOT4* regions. In the secondary non-coding variant analyses, multiple genes showed significant or suggestive associations with AD in LAT (Table 2B); however, no SVs were detected for those genes, and thus the observed associations were entirely driven by SNVs/INDELs. Not surprisingly, several of the associated genes overlapped with those identified in a previous study based solely on SNVs and INDELs, including *ELMSAN1*, *ACOT6*, and *ACOT4* [24].

In the candidate gene analyses, we examined 15 previously reported AD-associated genes to determine whether rare SVs contributed to their associations signals. No genes reached statistical significance in the primary analyses. In the secondary coding variant analyses, *PSEN1*, *TREM2*, and *ABCA7* showed evidence of association with AD (FDR Q < 0.05) (**Supplementary Table 8A**). Conditional analyses on the aggregates of SNVs/INDELs within each gene suggested independent effects of coding SVs for *PSEN1* and *ABCA7* (adjusted P-value < 0.05). Notably, *ABCA7* showed evidence of an independent SV association with AD, driven by a 605 bp exonic deletion (chr19:1050368–1050972) observed exclusively in four AD cases within AFR. This association remained significant after conditioning on disruptive missense SNVs/INDELs, indicating an independent effect of the deletion on AD risk. In the secondary non-coding variant analyses, *TREM2* and *ABI3* showed evidence of association with AD, however, no evidence of independent SV association with AD was observed (**Supplementary Table 8B**).

## 3. Discussion

In this study, we analyzed SVs in 16,841 individuals from ADSP using BioGraph, a novel SV calling pipeline. We identified 456,644 high-quality SVs, approximately 65% of which were novel. Notably, the vast majority of novel SVs were insertions, which may have been under-detected in previous studies. Among common or low-frequency SVs within each ancestry group, several SVs were found to be in moderate or high LD with known AD loci, offering additional insights into the genetic architecture of AD. Genome-wide association analyses identified two low-frequency deletions associated with AD in individuals of European ancestry, both located within genes encoding coiled-coil domain-containing proteins. Gene-based analyses further revealed that *PSEN1* and *ABCA7* harbor rare pathogenic SVs associated with AD.

Our use of BioGraph, a semi-assembly-based SV calling method, enabled the identification of many insertions not previously reported. Long-read sequencing and genome assembly studies have shown that insertions are the most prevalent SV class, often representing tandem repeat expansions or transposable element integrations that are not in the reference genome. This fact makes insertions challenging to identify, but also biologically intriguing as they have been reported to affect splicing or induce mosaic variants in proximity (e.g. ALUY insertions) [25]. Our SV call set demonstrated high accuracy and precision for both insertions and deletions, supported by rigorous benchmarking on replicate samples within ADSP and assessment across control samples. We have further introduced detection of inversions from BioGraph results that yielded multiple inversions candidates. This is noteworthy as the correct identification of inversions remains highly challenging [26].

We comprehensively assessed SVs and their potential impact on AD. Notably, many genes previously highlighted by SNV-based GWAS exhibited SVs either within the gene itself or within 5 kb. Overall, 95.3% of the postulated genes showed SV overlap. To further explore the role of SVs in established AD loci while accounting for ancestral differences, we examined SVs in known AD loci within each inferred ancestry group. We identified several SVs in moderate or high LD with known AD loci across different ancestry groups. At the *TPCN1* locus, a 319-bp intronic deletion was observed across the three ancestry groups in high LD with the tagging SNP. This deletion fully overlaps with a previously reported 309-bp deletion associated with Lewy Body dementia, which was validated using long-read sequencing [27]. *TPCN1*, which is highly expressed in the brain, encodes the two-pore calcium channel protein 1 located on endolysosomal membranes. Beyond its association with AD identified in previous GWAS [6], the function of *TPCN1* has been demonstrated in knockout mice, which exhibit impairments in spatial learning and memory [28]. In both EUR and LAT, we additionally discovered a deletion and an insertion at the *TPCN1* locus that showed evidence of association with AD in EUR. The deletion was located in *SLC8B1*, which encodes a mitochondrial Na+/Ca2 + exchanger. Notably, a recent study demonstrated that a deletion of the *SLC8B1* region in knockout mice, spanning the region of our four deletions, is sufficient to induce AD-like pathology, including age-related cognitive decline [29]. Our haplotype estimation analysis suggested that all detected SVs lie on the same haplotype as the AD risk allele at the *TPCN1* locus. These findings suggest that multiple genes within the *TPCN1* locus may influence AD risk and underscore the need for further investigation into the role of SVs at the *TPCN1* locus across ancestrally diverse populations and in other neurodegenerative diseases. *TMEM106B* encodes a transmembrane glycoprotein that localizes to late lysosome and endosome [30]. At the *TMEM106* locus, we detected a 322-bp Alu deletion in exon 8 or 3’ untranslated region, which has previously been reported as a likely causal variant and validated using long-read sequencing data [31]. This deletion has been associated with not only with AD, but also with frontotemporal lobar dementia with TDP-43 inclusions (FTLD-TDP) [31], neurodegeneration [32], and several AD-related phenotypes, including tangles density, TDP-43, and cognitive resilience [33].

From genome-wide analyses of common or low frequency SVs, we identified two significant deletions associated with AD among EUR. Both deletions are located in genes encoding CCDC proteins. Members of this family are characterized by an N-terminal potential microtubule binding domain, a central coiled-coiled and a C-terminal Hook-related domain. An 80-bp deletion on chromosome 11 is located in intron 7 of *CCDC88B* and encompasses *MIR7155* (chr11:64,341,849 – 64,341,904). *CCDC88B* has been shown to act as a positive regulator of T-cell maturation and inflammatory function [34]. The low frequency deletion on chromosome 3 is located in intron 1 of *CCDC12* and 2 Kb upstream of neurobeachin like 2 (*NBEAL2*). The functions of *CCDC12* remain unclear but it is predicted to be part of the spliceosomal complex. *NBEAL2* is thought to play a role in megakaryocyte alpha-granule biogenesis. In public databases, these two deletions are annotated as indels with rsIDs rs1553653356 (chr3) and rs1591274862 (chr11), respectively. In the gnomAD database (v4.1.0) [35], rs1553653356 shows a low frequency (AF = 0.002), consistent with our findings. In contrast, rs1591274862 (chr11) shows a notable discrepancy between the exome data (AF = 0.2) and genome data (AF = 0.002), although this variant failed quality control in both datasets. This discrepancy highlights the need for further investigation. Nonetheless, at the gene-level, a pQTL for *CCDC88B* and an eQTL for *CCDC12* have been previously associated with AD [36, 37], suggesting potential causal links between these genes and AD. Functional validation of the two deletions is warranted.

We identified multiple genes associated with AD in gene-based analyses of rare SVs and SNVs/INDELs, particularly in LAT. The Latino population is genetically admixed, with varying proportions of European, African, and Amerindigenous genetic backgrounds, which adds genetic complexity [38]. According to GrafPop, the Latin American 1 population primarily represents individuals with European and African ancestry components, whereas the Latin American 2 population mainly represents individuals with European and Amerindigenous components [39]. Notably, the Latin American populations exhibit unique LD patterns and haplotype structures derived from admixture [40], which may enhance the detection of rare variants. For *PSEN1*, we identified four rare, coding SVs with evidence of association with AD. All four SVs were annotated as highly pathogenic by indirectly altering *PSEN1* regulatory elements. Indeed, all four SVs are located in regions of neighboring genes, not within *PSEN1* itself, highlighting the impact of SVs through long range regulatory mechanisms [41]. For *ABCA7*, we identified a rare 605-bp deletion that partially overlaps intron 18 and exon 19, showing evidence of association with AD risk in AFR. This observation aligns with findings from a recent study [42], despite their use of a different SV caller and statistical model. This deletion is located approximately 3 kb downstream of a previously reported 44-bp deletion (rs142076058) associated with AD risk in African American individuals [43]. Additionally, it overlaps with a well-characterized SNP (rs115550680) previously associated with late-onset AD in African Americans populations [44]. In our dataset, all four individuals carrying the rare 605-bp deletion had AD and did not carry the previously reported deletion or SNPs associated with AD among African populations, whereas two common SNPs (rs3764650 and rs3752246) previously identified in European AD GWAS were observed [45]. Our findings provide new insights into the genetic architecture of the *ABCA7* locus in African ancestry populations. SPARC-related modular calcium-binding protein 1 (SMOC1) has consistently been reported as a biomarker for early AD in proteomics studies [36, 46–49], although the underlying genetic basis remains unclear. While we did not observe any rare SVs for *SMOC1*, the observed aggregate of synonymous SNVs/INDELs suggestively associated with AD in this gene may partially explain the genetic contribution to the increased levels of SMOC1 in AD [50].

We acknowledge several limitations in this study. Despite the relatively large sample of ascertained AD cases, statistical power remains limited, particularly for rare noncoding SV analyses within ancestry subgroups. Analyses using pooled populations did not yield additional associations for common SVs and may have introduced potential false positives in the gene-based analyses, possibly due to data structure complexities inherent to rare SVs and incomplete adjustment for population stratification using PCs. Another limitation is the lack of replication for our findings. While we identified two significant deletions associated with AD, their low frequency poses challenges for replication. Nonetheless, validation in larger and independent cohorts will be essential.

## 4. Conclusions

In conclusion, we identified high-quality SVs in ADSP samples using a novel SV calling method. Our analysis revealed ancestry-specific SVs at known AD loci, as well as both common and rare SVs associated with AD. These findings provide new insights into genetic architecture of AD. Future studies are warranted to validate our results and investigate the functional impact of these SVs.

## 5. Methods

### 5.1. Study samples

The Alzheimer’s Disease Sequencing Project (ADSP) was initiated in 2012 to elucidate the genetic architecture of AD, with major goals to identify genes and gene variants that confer risk for or protection against AD, to provide insight as to the biological impact of these genes and variants, and to identify potential therapeutic targets [51]. The WGS data release used in the present study (Release 3) includes data from 16,841 diverse individuals with and without AD from 24 cohorts. Raw data were obtained from the National Institute on Aging Genetics of Alzheimer’s Disease Data Storage Site (NIAGADS). After removing duplicates (Section 5.4), outliers subjects (Section 5.5), and subjects with missing phenotypic information, 12,908 samples (6,604 controls, 6,304 cases) remained for analysis.

### 5.2. SV detection

BioGraph version v6.0.4 was run per-sample using GRCh38 as the reference [16]. Variant Call Format (VCF) files were filtered to variant sites at least 50 bp long and with a PASS filter. Inversions were identified from all VCF entries with at least a 50 bp reference and 50 bp alternate allele reported where the sequence similarity of the reference and the reverse complement of the alternate allele was at least 80%. SVs were merged using bcftools v1.15 [52] and SVs with over 95% sequence and size similarity within 1000 bp were consolidated using Truvari collapse v3.1 with parameter – – *keep* max *qual* [21]. When necessary, SVs were cross-referenced to intersecting tandem repeat regions from the adotto TR catalog [53].

### 5.3. SV benchmarking using challenging, medically-relevant genes (CMRG)

We used WGS data from HG002, a sample with broad consent for open genomic data sharing through the Personal Genome Project [54]. SVs were called using BioGraph [16], Manta v1.6.0 [17], Parliament2 [18], and smoove 0.2.6 [19]. Truvari v3.5 [21] was used to compare the resulting SV calls against the CMRG benchmark [20]. Default truvari parameters were used for BioGraph and Manta. The parameters – – *dup* – → – ∈ *s* and – – *pctsim*0 were used for Parliament2 and smoove as neither tool produces sequence resolved calls.

### 5.4. Quality control of SVs using replicates analysis

Truvari v3.1 was run between 428 replicate pairs (ADSP participants with more than one sample sequenced). SV calls with over 70% sequence and size similarity between the replicates were classified as being consistent and the remainder classified as being inconsistent. Truvari annotations of PctSeqSimilarity and PctSizeSimilarity between consistent SV pairs were also analyzed to identify SVs that are the same across samples.

### 5.5. Quality control of samples using One-Class Support Vector Machine (SVM)

Passing SV counts by type were collected for each sample. Classification of the 2% of outlier samples by counts was performed using scikit-learn v1.1.3 and their OneClass SVM with hyper-parameters kernel=‘poly’ and nu = 0.02. Intersection of samples with the study which provided them showed a concentration of outlier samples from 3 of the 24 studies comprising the ADSP study sample. All samples included in these three studies (N = 421) were dropped.

### 5.6. Intersection with known SVs and annotation to genes

SVAfotate version 0.0.1 was used to intersect the discovered SVs with known SVs [22]. This program comprises an annotated file containing boundaries of SVs from 1000G [9], CCDG [55], and gnomAD [56]. TopMed SVs freeze 1.1 [57] were also collected and consolidated into the annotated file. SVs were annotated to genes with VEP using VEP-ensembl version 107.0 [58]. Next, we examined whether any of the discovered SVs mapped to genes reported by the ADSP Gene Verification Committee [59].

### 5.7. Global ancestry inference

Global ancestry inference of the study samples was performed using GrafPop [39, 60], a distance-based method that uses a reference composed of nearly 100,000 fingerprint SNPs extracted from dbGaP [61]. Grafpop estimates ancestry by calculating genetic distances between each individual and the reference populations, and subjects are clustered using genetic distances based on their genetic similarity. This tool considers that individuals’ genomes are admixed from three ancestries: European (E), African (F), and Asian (A) and estimates ancestral proportions P_e_, P_f_, and P_a_ based on genetic distances score using barycentric coordinates. In GrafPop, the cutoff thresholds were empirically defined to facilitate the grouping of dbGaP subjects. Due to the incompatibility of GrafPop with SV data, we used ADSP WGS data on single nucleotide variants (SNV) to perform global ancestry inference.

Using the cutoff standard established by GrafPop, ADSP participants with WGS data were clustered into nine groups defined by study-reported populations within dbGAP. These groups include European, African, East Asian, African American, Latin American 1, Asian-Pacific Islander, South Asian, Latin American 2, and Other, based on their ancestral proportions and genetic distance [39]. We grouped African and African American populations as African ancestry group (AFR) and Latin American 1 and Latin American 2 populations as Latin ancestry group (LAT), and European population as European ancestry group (EUR) [24]. Participants identified as East Asian, Asian-Pacific Islander, South Asian, and other populations were grouped as others and were excluded from subgroup association analyses due to limited sample size.

### 5.8. Principal Component Analysis

Principal component analysis (PCA) was performed using PC-AiR [62] in the GENetic EStimation and Inference in Structured samples (GENESIS) package [63]. We calculated PCs for all individuals (N = 12,908) in the study sample using high quality deletions, insertions, and inversions with minor allele frequency (MAF) greater than 1%, and with Hardy Weinberg Equilibrium (HWE) P-value greater than the Bonferroni-corrected threshold based on the total number of SVs (P = 5.2E-07). SVs in linkage disequilibrium (LD) were excluded using a r^2^ threshold greater than 0.1. For comparison with SNVs, we performed PCA on the same sample using biallelic SNPs with MAF > 1%, HWE P-value > 1E-06, and call rate > 95%.

For ancestry-specific SV association analyses, we calculated PCs using SVs with MAF > 1% and HWE P-values exceeding the Bonferroni-corrected threshold based on the number of SVs with MAF > 1% in each ancestry group (AFR: P = 4.7E-07; EUR: P = 6.5E-07; LAT: P = 5.4E-07). For ancestry-specific SNVs association analyses, we focused on previously reported AD GWAS SNPs that were in LD with our SV calls. For these analyses, PCs were calculated using WGS data filtered for MAF > 1%, HWE P > 1E-06, and call rate > 95% within each ancestry group.

### 5.9. SVs tagging known AD GWAS SNPs

To investigate the role of SVs in known AD GWAS loci, we examined 147 SNPs tagging AD loci identified in previous GWAS [6, 7, 23, 64, 65]. These variants were extracted from our WGS data, and we performed pairwise LD analysis between the AD-associated SNPs and our SVs within each ancestry group (Fig. 1A). Among common or low-frequency SVs with MAF > 0.5% and in HWE (see above), we specifically focused on SVs that were in LD (r^2^ > 0.4) with at least one of the AD-associated SNPs. LD calculation was carried out using PLINK v1.9 with parameters --ld-window-r2 0.4 and --r2. Haplotype estimation was performed using PLINK v1.9 based on pairwise LD patterns.

### 5.10. Association analyses

#### 5.10.1. Models and Covariates

Within each ancestry group inferred based on genetic similarity, association analyses were conducted using a mixed effects logistic regression model. Detailed models and software for common and rare SV analyses are provided in the corresponding sections below.

Covariates included sex, SV-derived PC 1–5 of each ancestry, relatedness via a genetic relatedness matrix (GRM), and technical covariates including sequencing center and whether the sample preparation was PCR-free.

For the analysis evaluating SVs in LD with SNPs tagging AD loci, we applied a conditional model that further included the corresponding SNP dosage. We also performed association analyses of the identified SNPs in LD with SVs, replacing SV PCs with SNP PCs, and including the corresponding SV dosage as a covariate in the conditional model.

#### 5.10.2. Single variant analysis of common or low-frequency SVs

Association analyses of common and low frequency SVs (MAF > 0.5% and passing the HWE criterion) were conducted using a mixed-effects logistic regression model implemented in the GENESIS R-package [39], with covariates and models as described above. All association analyses were performed within each ancestry group. For genome-wide association analysis, a meta-analysis was additionally performed across all ancestry subgroups using METAL software, implementing Stouffer method to weight results by sample size.

To identify AD-associated SVs, we considered several P-value thresholds: For evaluating SVs in LD with known AD loci (Fig. 1A), a suggestive significance threshold (P < 0.1) was used. For genome-wide analysis of all common or low-frequency SVs (Fig. 1B), two significance thresholds were considered: a Bonferroni-corrected threshold (AFR: P < 1.7E-06; EUR: P < 3.4E-06; LAT: P < 1.6E-06) based on the total number of SVs analyzed and the conventional genome-wide significance threshold (P < 5E-08).

#### 5.10.3. Gene-based analysis of aggregates of rare SVs

Gene-based association analyses of aggregated rare SVs with AD were conducted as the primary analyses. We included deletions, insertion, and inversion with MAF < 1% in each ancestry group and estimated their pathogenicity using PhenoSV [66]. PhenoSV is a machine learning based method that predicts the functional consequences of both coding and non-coding SVs that may directly or indirectly influence genes. SVs were classified as coding if they overlapped at least 1bp with exons of protein-coding gene based on GENCODE v40 annotations [67], considering only high-confidence representative transcript, otherwise they were classified as noncoding. Independently, SVs were evaluated for their potential to affect genes directly or indirectly. Non-coding SVs were tested for their indirect effect on genes within 1Mb upstream and downstream, as defined by default. Gene-level pathogenicity scores ranged from 0 to 1 and were used to classify SVs into pathogenic (≥ 0.5) and benign (< 0.5) groups [66]. Only rare pathogenic SVs were included in the analyses, which were performed separately for non-coding and coding variants (Fig. 1C, 1D).

In secondary analyses, we integrated SV data with SNVs/INDELs derived from ADSP 17K WGS data [24] to increase statistical power. The WGS data had been previously processed and quality-controlled according to the Genome Center for Alzheimer’s Disease (GCAD)/ADSP QC pipeline [68]. Using the WGS data annotated with FAVOR, we classified SNVs/INDELs as coding or non-coding based on the STAAR pipeline [69]. Variants with MAF < 1% within each ancestry group were included in the analysis. The coding SNVs/INDELs were categorized into five functional groups: putative loss of function (pLof), missense, disruptive missense, pLof + disruptive missense, or synonymous. The non-coding SNVs/INDELs were grouped into eight categories: promoter or enhancer overlaid with cap analysis of gene expression (CAGE) or DNase I hypersensitive site (DHS) sites, untranslated region (UTR), upstream, downstream, and noncoding RNA genes. Gene-based analyses were then performed within each category of coding and noncoding variants, combining rare pathogenic SVs of the corresponding type (Fig. 1E, 1F).

All gene-based association analyses were performed using the variant-set mixed model association test (SMMAT), implemented in the GMMAT R package [70]. We used a hybrid test (SMMAT-E), which combines burden and SKAT tests and has been shown to offer greater statistical power than either test alone. MAF was used as a weight by default. Genes with a cumulative minor allele count (cMAC) ≥ 10 were included in the analysis. All association analyses were stratified by ancestry group, and meta-analyses combining results across ancestry subgroups were conducted using the metap R package [71] with the Stouffer method to account for sample size differences.

We applied two significance thresholds for gene-based tests: Bonferroni-corrected threshold (P < 1E-07) to account for approximately 20,000 genes tested, and a suggestive threshold (P < 1E-05). For genes with significance in the secondary analyses, we performed conditional analyses on the aggregates of SNVs/INDELs within each gene to assess whether the association signal was driven by SVs.

Finally, we conducted candidate gene association analyses focusing on 15 AD genes previously reported to harbor rare variant associations [72, 73], evaluating them in both primary and secondary gene-based analyses. We computed the false discovery rate (FDR) Q value within each analysis group using the Benjamini-Hochberg procedure to assess statistical significance. For genes showing evidence of association with AD (FDR Q < 0.05) in the secondary analyses, we conducted additional conditional analyses on the aggregated SNVs/INDELs to evaluate the contribution of SVs to the observed signal.

## Supplementary Material

This is a list of supplementary files associated with this preprint. Click to download.

SupplementaryMaterial.docx

SupplementaryTables.xlsx

SupplementaryFigures.docx

## Figures and Tables

**Table 1:** Bonferroni-significant SVs associated with AD identified by genome-wide single variant association analyses

Group	SV type	SV locus	SV Breakpoints	Length	QUAL	Gene(s)	Location	Allele Frequencies			Single variant association tests	
								AFR	EUR	LAT	OR [95% CI]	P-value
EUR	DEL	3p21.31	46978504–46978622	119	89	*CCDC12*	intron	0	0.013	0	3.19 [2.1–4.9]	7.66E-08
EUR	DEL	11q13.1	64341844–64341923	80	94	*CCDC88B*	intron	0	0.011	0	2.89 [1.9–4.4]	2.09E-06

AFR, African ancestry group; LAT, Latin ancestry group; EUR, European ancestry group; DEL, deletion; QUAL, quality score; OR, odds ratio; CI, confidence interval

**Table 2:** Bonferroni-significant or suggestive genes associated with AD in gene-based association analyses of coding variants (A) and non-coding variants (B)

A. Coding SNVs/INDELs + SVs						
Group	Gene	Locus	Category	n variants	cMAC	P-value
LAT	*PSEN1*	14q24.2	pLof+disruptive missense	11	61	1.96E-07
LAT	*PSEN1*	14q24.2	missense	14	74	8.69E-07
LAT	*SMOC1*	14q24.2	synonymous	14	88	1.50E-06
LAT	*ACOT4*	14q24.3	disruptive missense	3	35	2.72E-06
LAT	*ACOT4*	14q24.3	missense	9	61	5.44E-06
LAT	*ACOT4*	14q24.3	synonymous	10	54	5.70E-06
B. Non-coding SNVs/INDELs + SVs						
Group	Gene	Locus	Category	n variants	cMAC	P-value
LAT	*ELMSAN1*	14q24.3	ncRNA	3	23	**1.86E-09**
LAT	*AC005225.2*	14q24.3	ncRNA	6	44	**6.61E-08**
LAT	*LOC100506476*	14q24.3	Promoter (CAGE)	21	93	**7.17E-08**
LAT	*LOC100506476*	14q24.3	Enhancer (CAGE)	26	103	1.03E-07
LAT	*ACOT6*	14q24.3	Promoter (DHS)	21	104	3.61E-07
LAT	*AL390763.1*	10q26.2	ncRNA	5	10	2.24E-06
LAT	*ACOT4*	14q24.3	Enhancer, Promoter (CAGE)	17	59	2.53E-06
LAT	*AC005225.2*	14q24.3	Promoter (DHS)	43	263	5.09E-06
LAT	*ACOT4*	14q24.3	Enhancer (DHS)	17	93	5.25E-06
LAT	*LINC01500*	14q24.1	Promoter (CAGE)	6	69	6.25E-06
LAT	*TIRAP*	11q24.2	UTR	6	16	7.43E-06
LAT	*PROX2*	14q24.3	Enhancer (DHS)	27	130	7.49E-06
LAT	*ACOT4*	14q24.3	Promoter (DHS)	19	103	9.08E-06
LAT	*AL163974.1*	14q32.2	Upstream	16	90	9.88E-06
LAT	*ACOT6*	14q24.3	Enhancer (DHS)	23	107	1.29E-07

SNV, single nucleotide variants; INDELs, insertion and deletions; pLof, putative loss of function; cMAC, cumulative minor allele count; ncRNA, non-coding RNA; CAGE, cap analysis of gene expression; DHS, DNase I hypersensitive site; UTR, untranslated region

## Data Availability

ADSP whole genome sequencing data (NG00067) are available through the National Institute on Aging Genetics of Alzheimer’s Disease Data Storage Site (NIAGADS) (https://www.niagads.org).
